# Effectiveness of bivalent COVID-19 boosters against COVID-19 mortality in people aged 65 years and older, Australia, November 2022 to May 2023

**DOI:** 10.2807/1560-7917.ES.2023.28.47.2300603

**Published:** 2023-11-23

**Authors:** Bette Liu, Sandrine Stepien, Ketaki Sharma, Kristine Macartney

**Affiliations:** 1 National Centre for Immunisation Research and Surveillance, Sydney Australia

**Keywords:** SARS-CoV-2, vaccine effectiveness, COVID-19 mortality, bivalent COVID-19 vaccine

## Abstract

We followed 4,081,257 Australian adults aged ≥ 65 years between November 2022 and May 2023 for COVID-19-specific mortality, when recombinant SARS-CoV-2 Omicron lineages (predominantly XB and XBB) as well as BA.2.75 were circulating. Compared with a COVID-19 booster targeting ancestral SARS-CoV-2 given > 180 days earlier, the relative vaccine effectiveness against COVID-19 death of a bivalent (ancestral/BA.1 or ancestral/BA.4-5) booster given 8 to 90 days earlier was 66.0% (95%CI: 57.6 to 72.2%) and that of a monovalent ancestral booster given 8 to 90 days earlier was 44.7% (95%CI: 23.9 to 59.7%).

There are limited data on how effective variant-specific COVID-19 vaccines are against COVID-19 mortality, particularly that caused by recombinant Omicron (Phylogenetic Assignment of Named Global Outbreak (Pango) lineage designation: B.1.1.529) lineages. We sought to compare the effectiveness of bivalent boosters, containing ancestral and Omicron-specific severe acute respiratory syndrome coronavirus 2 (SARS-CoV-2) antigens, and the effectiveness of monovalent ancestral COVID-19 boosters against COVID-19 mortality during the November 2022 to May 2023 period, leading up to the southern hemisphere winter when predominantly recombinant Omicron lineages (XB and XBB) were circulating in Australia [1].

## Data collection, estimations of death rates and relative vaccine effectiveness

We linked whole of Australian population census, migration, mortality, and Australian Immunisation Register data using methods described previously [2]. We followed 4,081,257 Australian adults aged 65 years and older from 1 November 2022 until 31 May 2023 for death from COVID-19 as specified on death registrations (underlying cause of death coded as International Classification of Diseases (ICD)-10 U07.1 or U07.2) using survival analysis. COVID-19 death rates were estimated according to vaccination status (vaccine type and recency of booster) and hazard ratios (HRs) were adjusted for age, sex (male/female), jurisdiction of residence, household income, number of comorbidities (based on a validated measure using individual-level pharmaceutical dispensing records over the previous 6 months before study entry) [3], number of general practitioner (GP) consultations in the year before study entry, and receipt of an influenza vaccine in 2022.

No individual identifying details were available in the databases and all results where counts or rates are presented were perturbed or suppressed according to the Australian Bureau of Statistics methods to prevent disclosure of small numbers and potential re-identification [4].

Relative vaccine effectiveness (rVE) as compared with a booster dose (> 180 days previously) was estimated using the formula rVE = (1 − adjusted HR) × 100%. 

## Characteristics of study population and vaccines received

At the start of the study period on 1 November 2022, the mean age of the 4,081,257 individuals included in the study was 74.8 years (standard deviation (SD): 7.4), 53.6% (n = 2,185,896) were women, 51.6% (n = 2,105,673) had four or more comorbidities, 77.8% (n = 3,174,537) had an influenza vaccine in 2022. Following completion of their primary vaccine course, most individuals had received at least one COVID-19 vaccine booster with 21.7% (n = 886,248) receiving one, 66.0% (n = 2,692,443) receiving two, and 0.9% (n = 36,484) receiving three boosters. Among those who had received a booster, 98.3% (3,554,248/3,615,175) had received the monovalent ancestral mRNA vaccine as the most recent booster type.

During the study period up to 31 May 2023, 73,250 died; 2,880 of COVID-19 and during this 7-month period, an additional 4% (n = 174,875) of the population received a second booster and an additional 35% (n = 1,431,623) received a third booster. Among all who received a booster and were followed to 31 May 2023 (n = 3,600,573), the most frequently received vaccine type for the latest dose was a monovalent ancestral mRNA vaccine (56.6%; n = 2,037,574) followed by the bivalent (ancestral/BA.4-5) mRNA vaccine (25.4%; n = 913,668) and bivalent (ancestral/BA.1) mRNA vaccine (16.8%; n = 605,043). Both the BioNTech-Pfizer (Mainz, Germany/New York, United States (US)) and Moderna (Cambridge, US) formulations were used. The vaccine type received by people differed by the booster number; among individuals who got a second booster, 88.7% (1,260,248/1,421,092) received the monovalent ancestral mRNA vaccine while among those who got a third booster, 57.6% (845,761/1,468,107) received the bivalent (ancestral/BA.4-5) mRNA vaccine and 35.6% (522,437/1,468,107) the bivalent (ancestral/ BA.1) mRNA vaccine.

## Relative vaccine effectiveness in preventing death from COVID-19

**Figure 1:**
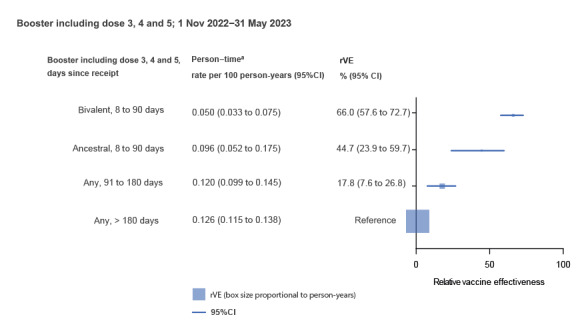
Effectiveness against COVID-19 mortality, of recent COVID-19 bivalent and monovalent ancestral booster vaccines relative to a booster vaccine given > 180 days earlier, Australia, 1 November 2022−31 May 2023 (n = 4,081,257 individuals)

Figure 1 shows rVE in preventing COVID-19 death of a booster dose given within the last 8 to 90 days (bivalent or monovalent ancestral) compared with a booster given > 180 days earlier (99.9% monovalent ancestral). The rVE was 66.0% (95% confidence interval (CI): 57.6 to 72.2%) for bivalent vaccines and 44.7% (95%CI: 23.9 to 59.7%) for the monovalent ancestral vaccine. For boosters given 91 to 180 days earlier (94.0% monovalent ancestral vaccine), rVE was 17.8% (95%CI: 7.6 to 26.8%).

95%CI: 95% confidence interval; rVE: relative vaccine effectiveness.

^a^ Person−time was 2,336,441 years.

There were 2,880 events of death by COVID-19 during the observation period.

**Figure 2:**
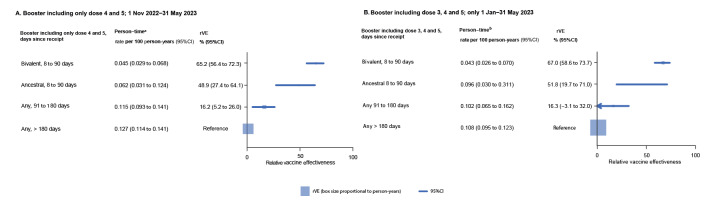
Effectiveness against COVID-19 mortality of recent COVID-19 bivalent and monovalent ancestral booster vaccines relative to a booster vaccine given > 180 days earlier, for (A) dose 4 and 5 boosters only and (B) follow-up restricted to 1 Jan−31 May 2023 period, Australia, 1 Nov 2022−31 May 2023

Sensitivity analyses are shown in Figure 2A and 2B. In the first, only individuals who received a second or third booster (corresponding to doses 4 and 5) are shown under the booster dose rVE estimates (those who received a first booster, dose 3, were classified separately) (Figure 2A); in the second, a shorter time interval (Jan–May 2023) was examined. Both analyses showed rVE was consistent with the main analysis.

95%CI: 95% confidence interval; rVE: relative vaccine effectiveness.

^a^ Person−time was 2,336,441 years. There were 2,880 events of death by COVID-19 during the observation period.

^b^ Person−time was 1,655,718 years. There were 1,730 events of death by COVID-19 during the observation period.

## Discussion

We found that in the Australian population aged 65 years and over, the majority of whom had already experienced a SARS-CoV-2 Omicron variant infection [5], bivalent mRNA COVID-19 boosters were likely to be more effective in preventing COVID-19 mortality than monovalent ancestral mRNA COVID-19 boosters during a period when recombinant Omicron lineages such as XB and XBB as well as BA.2.75, were circulating [1]. Recency of booster receipt also continued to be an important factor in mortality prevention with a lower rVE with increasing time since vaccine receipt.

Our findings add to the relatively scare literature on the effectiveness of variant-specific vaccines during a period of recombinant Omicron circulation. Previously published studies, as further described, have shown that both the bivalent ancestral/BA.1 or ancestral/BA.4-5 booster provide significant protection against severe illness from COVID-19. However, there are more limited data on whether the protection provided is greater than that from monovalent ancestral boosters.

A test-negative case–control study conducted in England during a period of Omicron dominance (September 2022–February 2023) reported that compared with vaccination more than 6 months ago in adults aged ≥ 50 years, the VE against hospitalisation of bivalent ancestral/BA.1 mRNA booster (both Moderna and BioNTech-Pfizer formulations) was 53% (95%CI: 47.9 to 57.5) at 2 to 4 weeks after administration, waning to 35·9% (95%CI: 31·4 to 40·1) after 10 or more weeks [6]. Similarly, a Nordic study conducted from 1 July 2022 to 10 April 2023 found a rVE of 70% (95%CI: 50 to 90%) against COVID-19 mortality of a bivalent booster (ancestral/BA.1 and ancestral/BA.4-5) as a fourth dose compared with a third dose but did not find this rVE substantially different from the rVE of a fourth monovalent ancestral dose compared to a third dose (rVE: 65%; 95%CI: 36 to 95%) [7].

A US study compared monovalent ancestral boosters administered during May to August 2022 with BioNTech-Pfizer or Moderna ancestral/BA.4-5 based boosters administered during September to December 2022, a period of BA.4.6, BA.5, BQ.1 and BQ.1.1 dominance. The differences in rVE of an additional booster between bivalent and ancestral monovalent vaccines were 33.5% (95%CI: 2.9 to 62.1) for protection against hospitalisation and 36.9% (95%CI: 12.6 to 64.3) for protection against death in the 15 to 99 days following administration [8]. Protection against symptomatic illness was more moderate when comparing bivalent with ancestral monovalent vaccines in a French cohort study conducted in adults aged ≥ 60 years, during October to November 2022. This study found that the BioNTech-Pfizer ancestral/BA.4-5 bivalent booster doses conferred an additional 8% (95%CI: 0 to 16) protection against symptomatic Omicron BA.5 infection compared with ancestral-based monovalent vaccines [9].

When it became available in Australia in November 2022, the ancestral/BA.1 bivalent COVID-19 booster was not preferentially recommended over monovalent ancestral vaccine. The ancestral/BA.4-5 bivalent booster was available from February 2023 and in March 2023 a third booster (5th dose for most) was recommended for all adults aged 65 years and older and some people with pre-existing conditions [10]. The timing of recommendations and vaccine availability meant that most individuals in our analysis who received a third booster received a bivalent vaccine, so separating the booster number from the vaccine type was not possible. However, earlier analyses in the Australia population suggest that time since receipt is more important than the booster number [2].

Study limitations include the lack of linked data on SARS-CoV-2 infection. Prior infection is known to decrease the risk of severe outcomes with reinfection [11]. We have also shown that those experiencing prior infection may be less likely to receive boosters [12]. However, for comparisons of booster type (bivalent vs ancestral monovalent) we used similar time intervals since booster receipt (8 to 90 days) compared with a common reference group (> 180 days). Confounding by previous infection would only arise if there were differences in the booster type received by prior infection status.

### Conclusion

As the SARS-CoV-2 Omicron recombinant variants continue to circulate in both the northern hemisphere autumn period and the southern hemisphere spring, our findings suggest that a recently administered new Omicron-variant specific booster can improve protection against COVID-19 mortality in older adults. Timely availability and preferential use of new variant-based vaccines, as recommended by World Health Organization [13] and others, appears to confer advantage and thus should be a priority in high-risk populations while recombinant variants continue to emerge.
